# Liver Fibrosis: From Basic Science towards Clinical Progress, Focusing on the Central Role of Hepatic Stellate Cells

**DOI:** 10.3390/ijms25147873

**Published:** 2024-07-18

**Authors:** Hikmet Akkız, Robert K. Gieseler, Ali Canbay

**Affiliations:** 1 Department of Gastroenterology and Hepatology, University of Bahçeşehir, Beşiktaş, Istanbul 34353, Turkey; 2 Department of Internal Medicine, University Hospital Knappschaftskrankenhaus, Ruhr University Bochum, In der Schornau 23–25, 44892 Bochum, Germany; rk.gieseler@gmx.de (R.K.G.); ali.canbay@rub.de (A.C.)

**Keywords:** hepatic stellate cells, myofibroblasts, macrophages, hepatocytes, liver sinusoidal endothelial cells, liver fibrosis, liver fibrosis resolution

## Abstract

The burden of chronic liver disease is globally increasing at an alarming rate. Chronic liver injury leads to liver inflammation and fibrosis (LF) as critical determinants of long-term outcomes such as cirrhosis, liver cancer, and mortality. LF is a wound-healing process characterized by excessive deposition of extracellular matrix (ECM) proteins due to the activation of hepatic stellate cells (HSCs). In the healthy liver, quiescent HSCs metabolize and store retinoids. Upon fibrogenic activation, quiescent HSCs transdifferentiate into myofibroblasts; lose their vitamin A; upregulate α-smooth muscle actin; and produce proinflammatory soluble mediators, collagens, and inhibitors of ECM degradation. Activated HSCs are the main effector cells during hepatic fibrogenesis. In addition, the accumulation and activation of profibrogenic macrophages in response to hepatocyte death play a critical role in the initiation of HSC activation and survival. The main source of myofibroblasts is resident HSCs. Activated HSCs migrate to the site of active fibrogenesis to initiate the formation of a fibrous scar. Single-cell technologies revealed that quiescent HSCs are highly homogenous, while activated HSCs/myofibroblasts are much more heterogeneous. The complex process of inflammation results from the response of various hepatic cells to hepatocellular death and inflammatory signals related to intrahepatic injury pathways or extrahepatic mediators. Inflammatory processes modulate fibrogenesis by activating HSCs and, in turn, drive immune mechanisms via cytokines and chemokines. Increasing evidence also suggests that cellular stress responses contribute to fibrogenesis. Recent data demonstrated that LF can revert even at advanced stages of cirrhosis if the underlying cause is eliminated, which inhibits the inflammatory and profibrogenic cells. However, despite numerous clinical studies on plausible drug candidates, an approved antifibrotic therapy still remains elusive. This state-of-the-art review presents cellular and molecular mechanisms involved in hepatic fibrogenesis and its resolution, as well as comprehensively discusses the drivers linking liver injury to chronic liver inflammation and LF.

## 1. Introduction

Chronic liver disease is the 11th leading cause of death, as well as the 14th leading cause of morbidity worldwide, and its global incidence continues to increase. Hepatic fibrosis is the common pathological mechanism resulting in cirrhosis and develops as a result of chronic liver injury from viral hepatitis, alcoholic liver disease, nonalcoholic fatty liver disease (NAFLD), or autoimmune liver diseases [1,2]. Liver fibrosis is a fibrous scar formation resulting from the excessive accumulation of extracellular matrix (ECM) proteins such as collagen and fibronectin, which is an important phase of tissue repair [1]. Upon liver injury, hepatic stellate cells (HSCs) become activated and increase the secretion of inflammatory mediators and the synthesis of ECM proteins; together, these alterations initiate the wound-healing process [2,3,4]. Minor and temporary tissue damage leads to a temporary increase in the accumulation of ECM proteins, which contributes to the healing of tissue damage [3]. However, if the injury is serious or recurrent, ECM proteins continue to accumulate, resulting in tissue architecture disruption and organ failure [3]. Fibrosis develops years or even decades after extensive and persistent liver damage and, intriguingly, is associated with a potent immune response [4]. It is characterized by the accumulation of collagen and other fibrillar proteins such as elastin in the space of Disse, as well as an increase in ECM proteins [4]. Importantly, excessive ECM production disrupts the liver’s architecture, which impairs liver functions, alters intrahepatic blood flow, and can progress to liver cirrhosis [5]. Transforming growth factor-β (TGF-β) is the central mediator responsible for the imbalance between ECM and degradation of liver fibrosis [2,3,4,5]. This is a very important detail, as TGF-β not only is an evolutionarily ancient and therefore fundamental regulatory element, but it also acts as a key factor of the termination phase in the context of liver regeneration [2,3,4]. Here, TGF-β inter alia inhibits the proliferation of hepatocytes and induces apoptosis [2,3,4,5]. These aspects may be likewise important in the development of liver fibrosis, so that the gentle modulation of TGF-β might serve as an antifibrotic option. Earlier, Xu et al. came to a similar conclusion, but they rightly pointed out that such an approach could, however, increase intrahepatic inflammation [6]. This dilemma therefore has to be addressed in the future.

In chronic liver disease, a close and potent relationship between inflammation and fibrosis has been proven [2,5]. Inflammation, the hallmark of chronic liver disease, drives progressive ECM deposition over time, which, in the long term, can progress to liver cirrhosis, end-stage liver failure, and liver cancer [4,5]. In contrast, successful antiviral treatment in patients with chronic viral hepatitis or with considerable lifestyle changes in NASH patients modulate the resolution of fibrosis [6,7,8,9]. These clinical observations suggest that hepatic fibrosis is a dynamic process in which inflammatory mechanisms play a dual role; on the one hand, they initiate and maintain fibrogenesis, while, on the other hand, they promote fibrolysis and fibrosis resolution [5]. Activated HSCs, as well as resident immune cells, promote the recruitment of ECM proteins within the sinusoids, leading to circulatory disorders [4]. In fact, HSCs are the main effector cells during hepatic fibrogenesis [2,5]. 

**Figure 1:**
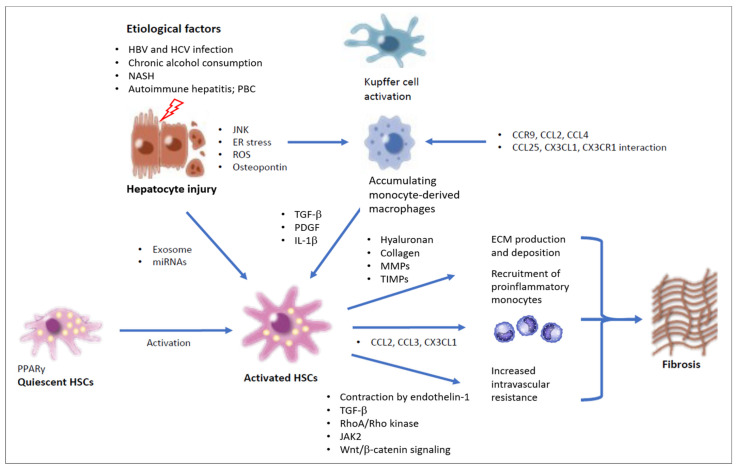
Overview of the cellular and molecular mechanisms of liver fibrogenesis. During chronic liver injury, hepatocytes activate signaling via Janus kinase (JNK), Notch, osteopontin, and hedgehog and produce exosomes harboring microRNAs (miRNAs) to initiate HSC activation. Inflammation triggers KCs and recruits monocyte-derived macrophages through C-C motif chemokine receptor (CCR)9 and C-C motif chemokine ligand (CCL)2, CCl_4_, and CCL25. The crosstalk between C-X3-C motif chemokine ligand 1 (CX3CL1) and C-X3-C motif chemokine receptor 1 (CX3CR1) orchestrates macrophage survival, differentiation, and polarization. KCs trigger the HSC activation by TGF-β, PDGF, and IL-1-β. Activated HSCs produce ECM proteins and secrete inflammatory chemokines CCL2, CCL3, and CX3CL1, whereby accumulating proinflammatory monocytes. HSC-originated matrix metalloproteinase (MMP) and tissue inhibitor of metalloproteinase (TIMP) contribute to ECM perpetuation, remodeling, and fibrosis. Activated HSCs lead to portal hypertension by enhancing the hepatic sinusoids’ contractility. Some molecules and pathways, including endothelin 1, TGF-β, Jak2, and the Wnt/β/catenin pathway, affect sinusoidal contractility.

Hepatotoxic and cholestatic liver injury are the major mechanisms of liver fibrosis [4,10,11]. Hepatotoxic injury results from the chronic impairment and damage of hepatocytes in conjunction with hepatitis B (HBV) or hepatitis C (HCV) infections, alcohol, or NAFLD [10,11]. Cholestatic injury occurs as a result of stasis in the bile flow, such as in primary biliary cholangitis (PBC), primary sclerosing cholangitis (PSC), and biliary atresia [12]. Studies in patients with liver fibrosis and in preclinical mouse models of liver fibrosis have documented the major molecules and mechanisms involved in fibrogenesis, i.e., the accumulation of bone marrow-derived inflammatory immune cells, secretion of proinflammatory cytokines, production of TGF-β by profibrogenic macrophages, activation of collagen type I (COLIAI)-secreting hepatic myofibroblasts, excessive production of the ECM, and the formation of a fibrous scar [4]. Recent data from patients with chronic liver disease and murine models have revealed that liver fibrosis can regress even at later stages of cirrhosis [1,2,4,5,13]. Following removal of the underlying etiological factor, liver fibrosis reverts, going along with the decreased production of proinflammatory and profibrogenic cytokines, upregulated collagenolytic activity, the disappearance of fibrogenic cells, decreased ECM production, and, eventually, the dissolution of the fibrosis scar [1,2,4,5,13,14,15,16,17]. Activated HSCs are eliminated by a bouquet of options that include not only apoptosis and cellular senescence but also reversion to the inactivated HSC phenotype [4,5,16]. Senescent HSCs are α-SMA-positive cells, but they neither proliferate nor produce collagen [2,5,10,11]. During acute inflammation, matrix metalloproteinase (MMP) is released to degrade ECM proteins to attenuate the infiltration of immune cells [2,4,5]. Once inflammation subsides, phagocytes clear the debris and release anti-inflammatory mediators [2,4,5]. This process results in the recruitment of endothelial cells, blood vessel growth, and activation of Kupffer cells (KCs) and myofibroblasts to reestablish the ECM, all of which enable the progression of wound healing [2,4,18]. An overview is provided in Figure 1.

## 2. Cell Types in Hepatic Fibrosis

Various cell types play key roles in the pathogenesis of liver fibrosis, including hepatocytes, inflammatory cells, liver sinusoidal endothelial cells (LSECs), and HSCs [2,3]. In response to liver injury, hepatocytes produce factors such as NOTCH, osteopontin, TGF-β, NADPH oxidase 4 (NOX4), the transcription regulator TAZ (a.k.a. WWTR1), and Indian Hedgehog protein. These factors initiate an inflammatory response, which results in macrophage activation, the secretion of reactive oxygen species (ROS) and TGF-β, and the transition of quiescent HSCs into myofibroblasts [4,5].

### 2.1. Hepatic Stellate Cells and Myofibroblasts

Recent technological advances such as single-cell RNA sequencing (sc-RNA-seq), high-dimensional multiomics (proteomics and genomics), and spatial transcriptomics have provided extensive new insight into the molecular–biological processes and the cell types involved, which, in turn, have led to a better understanding of the tissue biology and disease mechanisms in liver fibrosis [3].

HSCs are a nonparenchymal cell population that, in the healthy organ, accounts for around 5–8% of all liver cells [2,3]. HSCs are localized in the space of Disse, between the basolateral region of hepatocytes and the antiluminal surface of the liver sinusoidal endothelial cells (LSECs) [2,3,4]. In the healthy liver, quiescent HSCs store retinoids (vitamin A and its metabolites) within their cytoplasmic lipid droplets, which are essential for the perpetuation of quiescent HSCs [2,3,4,5]. 

Upon liver injury, the amount of vitamin A stored by quiescent HSCs decreases progressively, and the cells transdifferentiate into activated HSCs that produce αSMA, collagen I, inhibitors of matrix degradation, and proinflammatory cytokines, as well as chemokines that, by positive chemotaxis, attract immune cells to the region of inflammation [2,3,4,5]. Activated HSCs display a contractile, proliferative, and fibrogenic phenotype [3,4]. Ultrastructurally, they are characterized by a rough endoplasmic reticulum and a Golgi apparatus producing collagen [2,3]. Activated HSCs promote the recruitment and infiltration of immune cells in the liver by producing several proinflammatory cytokines, such as CCL2, CCL5, IL8, and chemokine (C-X-C motif) ligand-12 (CXCL12), and by expressing adhesion molecules, such as intercellular adhesion molecule 1 (ICAM-1) and vascular cell adhesion molecule 1 (VCAM-1) [2,4,5]. These cells migrate to the region of injury to contribute to the formation of a fibrous scar. HSCs, together with LSECs and hepatic macrophages, constitute the liver microcirculatory microenvironment, which plays a crucial role in governing the vascular tone and in controlling inflammation [5]. 

Experimental mouse models have revealed the existence of different subsets of HSCs that display distinct functions during chronic liver disease [2,4]. Single-cell technologies have shown that quiescent HSCs account for a relatively homogeneous population. However, activated HSCs can be discerned into several subsets [2,3,4,5,19,20]. Functionally, quiescent HSCs produce high levels of growth factors and can protect hepatocytes from injury, whereas myofibroblasts mainly express ECM proteins, such as collagen I; collagen III, VI, and XIV; cytokines; and chemokines that drive inflammation and fibrosis [2,3,4,5]. In cirrhotic livers, a population of quiescent HSCs has been detected that expresses various growth factors and cytokines and suppresses liver inflammation and fibrosis [2,5]. In addition, activated HSCs also display immunoregulatory functions: in different mouse models, they were found to promote T-cell apoptosis through PD-1 and PD-L1, affect B-cell function via immune checkpoint molecules, and contribute to immune tolerance through promoting immunosuppressive cells such as Tregs and myeloid-derived suppressor cells (MDSCs) [3]. At present, it is not clearly understood under which conditions HSCs act either proinflammatorily or tolerogenically [2,3]. There is an extensive interplay between HSCs and immune cells, such as macrophages [2,3,4,5]. Experimental trials using HSCs and macrophages have revealed that soluble mediators derived from activated HSCs promote the differentiation of macrophages into a proinflammatory phenotype [2,3] Additionally, novel data from scRNA-seq mouse studies reveal that HSCs communicate with the surrounding endothelial cells immune cells via soluble mediators, many of which are upregulated during liver injury [2]. 

Various factors, such as toxic lipids, lipid mediators, inflammation signals, growth factors, and distinct signals, including cell–cell contact with macrophages, cytokines derived from activated immune cells, and pathogen-associated molecular patterns (PAMPs) or danger-associated molecular patterns (DAMPs), mediate the activation of HSCs during liver injury [2,3,4,5,14,15]. Growth factors, including TGF-β, platelet-derived growth factor (PDGF), and epidermal growth factor (EGF), are key drivers of HSC activation [2,4,5,15,16,17]. Once activated, HSCs can release autocrine signals that maintain their fibrogenic state [16,17]. TGF-β is the central mediator in the activation of HSCs, which is produced by infiltrating lymphocytes and monocytes, KCs, and by damaged hepatocytes [2,4,5,19]. In liver fibrosis, this mediator is responsible for the imbalance between ECM synthesis and degradation [20]. In chronic liver disease, hepatocyte death and the resultant recruitment of macrophages play important roles in HSC activation and fibrosis via producing TGF-β [16]. In addition, the engulfment of cell debris by macrophages fosters their expression of TGF-β [16]. Macrophages remove apoptotic hepatocytes through the process of efferocytosis, which promotes the expression of growth factors, such as macrophage-derived TGF-β, resulting in the activation of HSCs [21] (Figure 1). The transition of quiescent HSCs into their activated phenotype is an energy-consuming process that requires fundamental reprogramming of their metabolic pathways [2,5]. Activated HSCs oftentimes display high-level expression of ECM-related genes [5]. Phosphorylation of SMAD proteins, e.g., SMAD3, mediates TGF-β-related HSC activation, which eventually results in the upregulation of collagen I and III synthesis [22,23]. In addition, α-SMA and connective tissue growth factor (CTGF) genes are upregulated by SMAD proteins [24]. TGF-β may promote HSC activation by other mechanisms besides the SMAD pathway, such as mitogen-activated protein kinase-1, p38, and c-jun N-terminal kinase-related mechanisms [25,26]. The type 3 cytokine, IL-17 derived from T-helper 17 (Th17) cells and neutrophils, exhibits an important role in activating the TGF-β signaling pathway [27]. In addition to this direct impact, latent TGF-β accumulated in the ECM is activated by the integrin-αv-mediated contraction of HSCs [27]. Therefore, deletion of the αv subunit in HSC prevents TGF-β activation and protects mice from liver fibrosis [2]. TGF-β signaling comprises various pathways, such as the non-canonical TAK1/JNK and the RAS/RAF/MAPK pathways, as well as the SMAD pathway, resulting in ECM synthesis [28,29]. TGF-β induces autophagy by interacting with the insulin-like growth factor-binding protein-associated protein 1, which activates the P13K/AKT/mTOR pathway [30]. In addition, the epigenetic regulator TET3 is involved in the TGF-β/SMAD2/3 activation pathway [2].

TGF-β-induced HSC activation is associated with an increased expression of other profibrogenic growth factors, including platelet-derived growth factor (PDGF) and vascular endothelial growth factor (VEGF) [20,31]. PDGF is a mitogenic growth factor which polypeptide chains A and B are arranged in AA, AB, or in BB combinations. Autocrine PDGF-BB stimulation is most potent to induce HSC proliferation [32,33]. As such, the autocrine stimulation of HSCs by TGF-β and PDGF-AA initiates HSC activation and perpetuates liver fibrosis [20,31,34]. PDGF acts on HSCs through PDGF receptor-β (PDGFR-β), which can promote increased signaling and HSC activation [34]. In HSCs, PDGFR-β activation induces their proliferation and migration, drives cell survival, and promotes the expression of hedgehog pathway ligands, such as sonic hedgehog, which triggers HSC activation [35,36]. Interestingly, experimental PDGFR-β deletion resulted in the regression of liver fibrosis, whereas the activation of this receptor enhanced fibrogenesis [37,38]. In line with this finding, there is a correlation between the degree of fibrosis and the PDGFR-β level in patients with NAFLD [39]. Under physiological conditions, PDGF is mainly expressed by platelets, while the factor is additionally expressed by endothelial cells, macrophages, and activated HSCs in liver diseases [2,38,39]. During liver injury, VEGF is produced by hepatocytes, which promotes HSC activation and proliferation, leading to an increased production of ECM proteins and TGF-β. Similarly, the accumulation of cholesterol and fatty acids during metabolic liver injury leads to the release of hedgehog ligands and exosomes that promote HSC proliferation and ECM production [5]. The phagocytosis of cholesterol-laden hepatocytes by KCs and macrophages differentiated de novo from infiltrating monocytes causes inflammasome activation and the production of proinflammatory cytokines, which perpetuate liver inflammation, HSC activation, and fibrosis [5].

Studies on the origin of hepatic myofibroblasts have documented their heterogenous derivation, which inter alia includes HSCs, portal fibroblasts, and bone marrow-derived cells such as fibrocytes and mesenchymal stem cells [40,41,42]. In contrast, liver parenchymal cells do not serve as a source of myofibroblast generation [4,40]. Recent studies have clearly identified both liver resident-activated HSCs and activated portal fibroblasts to be the main sources of hepatic myofibroblasts in chronic liver disease [43,44]. This view is strongly supported by results from cell fate mapping and deep phenotyping in both hepatotoxin-induced liver fibrosis models and models of biliary fibrosis, where activated HSCs and activated portal fibroblasts were found to account for more than 90% of collagen-producing cells [43,44]. However, the composition of myofibroblasts is highly variable, depending on the underlying cause of liver fibrosis. HSCs are usually activated as a result of toxic liver injury affecting the centrilobular and perisinusoidal regions of the liver. However, both activated HSCs and portal fibroblasts promote cholestatic liver fibrosis caused by periportal injury [43,44]. In biliary fibrosis models, 70% of the myofibroblast population are derived from activated fibroblasts at the onset of cholestatic injury, while the majority of myofibroblasts in later stages of the disease was found to originate from HSCs [43]. In addition, fibrocytes constitute a small population of bone marrow-derived myofibroblasts in mice with cholestatic or toxic fibrosis [45]. Potential other sources of hepatic myofibroblasts in the pathogenesis of liver fibrosis might include epithelial-to-mesenchymal transition (EMT) [46], but, at least in mice, cell fate mapping studies excluded EMT from the pathogenesis of liver fibrosis [47,48,49,50,51]. It has been reported that mesenchymal stem cells experimentally obtained from murine bone marrow proliferate in the liver and lead to an increase in tissue fibroblasts [52].

Differentiation of quiescent HSCs into activated HSCs is a two-step process: The initiation phase encompasses the priming of HSCs, which sensitizes them to fibrogenic and proliferative cytokines [41,47,48,53]. The initiation of liver fibrogenesis is accompanied by increased cell activity containing the proto-oncogene c-myc, as well as cyclin E1 [23]. Damaged and apoptotic hepatocytes induce HSC activation by degrading the regular ECM composition in the space of Disse, releasing reactive oxygen species and proinflammatory cytokines and recruiting immune cells, which collectively perpetuates HSC activation [54,55,56,57]. Activation of the PDGF receptor, the occurrence of a contractile and fibrogenic phenotype, and alteration of growth factor signaling are the fundamental properties of the initiation phase [53,58]. During the initiation phase, quiescent HSCs transdifferentiate into activated HSCs that produce PDGF and PDGFR-β. PDGF promotes HSC proliferation and results in the increased production of profibrogenic cytokines such as TGF-β, which further activate HSCs to upregulate α-SMA expression and stimulate ECM secretion [2,3]. Accordingly, in animal models of experimental liver fibrosis, depletion of PDGFR-β causes a reduction in fibrosis, while autoactivation of this receptor increases fibrogenesis [2,3]. In addition, levels of circulating PDGFR-β can provide information about the extent of liver fibrosis [2]. The perpetuation phase is characterized by specific phenotypic alterations, including proliferation, contractility, fibrogenesis, altered matrix degradation, and inflammatory signaling [2,4,53,59]. The composition, distribution, and amount of ECM proteins in fibrogenesis vary depending on the etiological factor. In the healthy liver, the ECM in the space of Disse predominantly consists of collagen IV and laminin [2,3,58]. During chronic injury, fibrillar collagens such as collagen I and III become essential proteins of the ECM [58]. Elimination of the underlying cause leads to resolution, during which HSCs undergo apoptosis, become senescent, or revert to an inactive HSC phenotype [2,4,53].

### 2.2. Inflammatory Cell Species Driving Hepatic Fibrogenesis

Chronic inflammation plays a critical role in the pathogenesis of hepatic fibrosis [2,4,5,59]. The immune response in the acutely damaged liver can be initiated by endogenous molecules [59]. Damaged and dying cells release various soluble molecules acting as DAMPs. Necrotic cells release high mobility group box 1 protein (HMGB1) into the microenvironment by necrotic cells. However, macrophages and dendritic cells (DCs) can contribute as well [59], and, in fact, HMGB1 recruits these myeloid cells to the damaged site. In the early stages of chronic liver disease, damaged parenchymal cells release inflammatory cytokines and soluble mediators that induce the activation of inflammatory cells, which include macrophages, lymphocytes, and NK cells [2,5,59]. Neutrophils are critical first responders of the innate immune system, and they can contribute to hepatic inflammation by producing proinflammatory cytokines and neutrophil extracellular traps (NETs), activating KCs, and recruiting additional types of immune cells [60,61,62]. Upon liver injury, neutrophils are rapidly recruited to the injured site to remove the apoptotic cells [61]. These cells further serve as a source of cytokines, and they kill bacteria by ejecting nuclear and mitochondrial DNA nets and by releasing antimicrobial enzymes [4,62]. In mice, the elimination of neutrophils or the deletion of neutrophil-derived soluble molecules (IL-8, IL-18, IL-17, CCL3, CCl_4_, and CCL2) attenuates the development of hepatic fibrosis [63,64]. Damaged and dying hepatocytes release P2Y_14_ ligands, such as uridine 5′-diphosphate (UDP)-glucose and UDP-galactose, which, on HSCs, bind to the P2Y_14_ receptor and promote HSC activation [5,65]. In mice, an experimental P2Y_14_ deficiency led to reduced fibrosis [65]. In addition, damaged hepatocytes may release nuclear HMGB1 and directly activate HSCs [66]. Mitochondria-derived danger signals (mito-DAMPs) are abundant in the liver, as hepatocytes comprise a high number of mitochondria due to their high metabolic activity [67] (see also below). Thus, mito-DAMPs are increased in patients with chronic liver disease and have been evidenced to stimulate HSC activation and scar tissue formation in experimental mouse models [68]. Some liver cell populations, including HSCs, KCs, and LSECs, express receptors for these danger signals [68]. The formation of an inflammasome is a critical process that triggers the inflammatory response via IL-1-β and IL-18, which eventually induces inflammatory cell death [69].

Macrophages play central roles in the maintenance of liver homeostasis, tissue remodeling, and the induction of immune responses [2,4,70]. They play a critical role during inflammation, injury, and fibrogenesis, but they can also promote fibrosis resolution [5,70]. Hepatic macrophages, which, in the healthy liver, consist exclusively of yolk sac-derived KCs, are increasingly displaced by bone marrow-derived macrophages in the diseased liver [70]. They display high phenotypic and functional diversity and plasticity, which means that they change their transcriptional profiles and functions based on environmental, tissue-associated, and inflammatory stimuli [5,70,71,72]. KCs are the major cell population of the innate immune system; this particular cell population is able to sense hepatocyte stress and injury signals from other cells, as well as to engulf cellular debris and release inflammatory signals [5,73]. The activation of TGF-β-expressing macrophages plays an important role in hepatic fibrogenesis [2,4,5,70]. Generally, liver injury activates liver macrophages that thereupon phagocytose bacteria and remove their metabolites from the portal circulation [5,70]. Activated hepatic macrophages produce various cytokines, including TGF-β1, PDGF, TNF-α, IL-1, IL-6, IL-10, CXCL1, CCL2, and CCL5 (also known as RANTES), and mediators such as ROS that trigger HSC activation and drive the infiltration of bone marrow-derived monocytes and neutrophils into the damaged liver [5,70]. Monocyte-derived macrophages and KCs are the main sources of TGF-β. 

Macrophages perpetuate the survival of myofibroblasts via IL-1-β and TNF-dependent activation of NF-κB [2,3,4,5,70]. CCL3 is a ligand for CCR1 and CCR5 that fosters the progression of hepatic fibrosis [4,70]. In mouse models of liver fibrosis, induced by CCl_4_ or by a methionine- and choline-deficient diet, blocking CCL3 or CCL5 decreased HSC activation and reduced fibrosis [2,4,5]. Genetic deletion or inhibition of myeloid TGF-β improved liver fibrosis in mice, whereas genetic overexpression of TGF-β promoted liver fibrosis, indicating that TGF-β is a key driver of fibrosis [74,75,76]. However, long-term depletion of TGF-β may cause impaired liver regeneration, delay wound healing, and may increase carcinogenesis [77]. IL-6, TNF, and IL-1-β may act synergistically with TGF-β, and the genetic deletion of these cytokines may reduce the development of liver fibrosis [78,79]. Lipopoly saccharide (LPS), IL-1-β, and TNF can enhance TGF-β signaling by downregulating BMP activin membrane-bound inhibitor (BAMBI), which serves as a pseudo-receptor for the TGF-β type I receptor family and a negative modulator of TGF-β signaling [80,81]. TGF-β and IL-6 are key drivers in the differentiation of naïve T cells to Th17 cells [79,82]. Murine Th17 cells express remarkably potent profibrogenic IL-17A and anti-inflammatory IL-22 [5]. Studies using single-cell technologies revealed that monocyte-derived macrophages can replace KCs and acquire a phenotype of lipid-associated macrophages or scar-associated macrophages (SAMs), including TREM2, CD9, and osteopontin expression [83,84,85]. Lipid-associated macrophages can be subdivided into a transitional CX3CR1^+^CCR2^+^ lipid-associated subpopulation and classical trem2^+^Cd3^+^Cd9^+^Gpnmb^+^ lipid-associated macrophages [70,86,87]. Their function is associated with the expression of triggering receptor (TREM2), which mediates lipid uptake and metabolism [87].

Macrophages have a key role during inflammation, injury, and fibrogenesis, but they can also support the resolution of fibrosis [88]. During the progression of fibrosis, inflammation accelerates macrophage trafficking and promotes their accumulation in the liver, where these cells express cytokines and chemokines that stimulate HSC activation [88]. The monocyte influx into the liver is mainly regulated by the chemokine monocyte chemo attractant protein-1 (MCP-1; a.k.a. CCL2) and its associated receptor C-C chemokine receptor 2 (CCR2) [88]. The MCP-1/CCR2 pathway is particularly activated in NASH patients [70]. KC- and HSC-derived CCL2 contribute to the increased differentiation of immature monocyte-derived LYC6C^hi^ macrophages in the liver [89]. Macrophages play a critical role in the resolution of liver fibrosis and are the main source of fibrinolytic matrix metalloproteinases (MMPs), including MMP12 and MMP13 [70]. During the regression of liver fibrosis, macrophages differentiate into a LY6C^low^ phenotype, stop the production of proinflammatory and fibrogenic factors, and secrete MMPs [90]. Macrophages also produce MMP9 and TNF-related apoptosis-inducing ligands (TRAIL) that promote HSC apoptosis [74]. A CD11b^hi^/F4/80^inf^LY6C^low^ macrophage subpopulation was identified during the resolution of liver fibrosis; these special cells do not produce fibrogenic and/or inflammatory factors, but they continue to secrete MMPs, including MMP9 and MMP12 [59,74,89], and they upregulate CX3CR1. Compared to wild-type mice, mice deficient in CX3CR1 are associated with increased inflammatory cell accumulation and fibrosis after CCl_4_ treatment [74]. Mechanistic studies, combining RNA sequencing, functional in vivo studies, and coculture experiments, have revealed that mucosal-associated invariant T-cell (MAIT) monocyte/macrophage interplay promotes liver fibrosis regression via reprogramming the macrophage phenotype [13]. MAIT cells recruited to the fibrotic septa of the diseased liver exhibit an activated phenotype, which directly impacts hepatic myofibroblasts and can contribute to the progression of fibrosis by enhancing a local inflammatory response [13].

Liver inflammation is also strongly driven by extrahepatic signals originating from other organs such as the gut or adipose tissue [5]. In chronic liver disease, the gut–liver axis, which refers to the anatomical and physiological connection between the liver and gut, is influenced by dietary fat and protein, bacterial metabolites, PAMPs, and intestinal and adipose tissue hormones; this may promote the progression of liver disease [91]. Also, the intestinal microbiome influences hepatic bile acid metabolism and the translocation of gut-derived signals due to leaky gut; these parameters may be found to be predictive of clinical outcomes in patients with chronic liver disease [92,93,94]. In NASH, the gut–liver axis is activated, and the interaction between liver injury, liver regeneration, and increased gut permeability may foster inflammatory, profibrogenic, and procarcinogenic pathways [93]. As a result of the gut’s permeability defect, the intestinal microbiome and the liver interact through bacteria and bacterial metabolites, which affect liver metabolism and further drive hepatic inflammation [95]. The gut permeability defect leads to the translocation of microbial products such as LPS, which elevates their serum levels and promotes the proinflammatory response elicited by hepatic macrophages [96]. In addition, dysfunctional gut permeability enables PAMPs and DAMPs to enter the liver where they activate immune cells and engage with hepatic Toll-like receptors (TLRs) to induce proinflammatory and fibrotic pathways [97,98]. Furthermore, the gut microbiome has a role in controlling the composition of bile acids and modifying secondary bile acids before being recycled to the liver [99]. Bile acids, such as cholic and chenodeoxycholic acid (CDCA), are important signaling molecules and are synthesized from cholesterol in hepatocytes surrounding the hepatic central vein [100]. Their synthesis is regulated by the farnesoid X receptor (FXR) mainly via downstream targets [101]. If low levels of primary bile acids reach the small intestine, proinflammatory bacteria proliferate and the production of toxic bile acids increases, which promotes liver damage and inflammation [102]. Conversely, hepatocellular FXR activation by different bile acids prevents the upregulation of inflammatory response genes and induces cell survival [103]. Therefore, the composition of the bile acid pool is an important determinant in the clinical outcomes of liver inflammation and fibrosis, which are closely related to gut microbiome dysbiosis [103]. In metabolic liver disease, free fatty acids flowing from adipose tissue to the liver may promote lipotoxicity and inflammation, particularly in patients with insulin resistance [104]. Furthermore, experimental trials have revealed that adipose tissue-derived leptin activates KCs and increases their responsiveness to endotoxin, which results in inflammation [105,106]. Free fatty acids and ethanol can also drive liver inflammation.

### 2.3. Hepatocytes

Hepatocytes represent approximately 80% of the liver’s total cell number [4,5]. Under physiological conditions, they have several functions, such as biotransformation/detoxification and the production of bile, as well as numerous proteins and lipids [4,5]. Hepatocytes are also a primary target for toxic metabolites influencing the liver [2,4,68]. Regardless of the underlying etiology, liver injury damages hepatocytes, which thereupon release intracellular molecules such as DAMPs that drive chronic inflammation and fibrogenesis [68]. These molecules are recognized by the innate immune system via pattern recognition receptors that oftentimes are the same molecular sensors that detect pathogens [68]. Due to their critical metabolic function, hepatocytes are abundantly furnished with mitochondria, with a stunning number of 1000–2000 of these organelles per hepatocyte [107]. Mito-DAMPs released from damaged hepatocytes, including adenosine triphosphate (ATP), DNA fragments, and fatty acids, promote the recruitment of resident and infiltrated macrophages, neutrophils, and NK cells [68,108]. In fact, mito-DAMPs derived from damaged hepatocytes may be among the most abundant and potent danger signals perpetuating the innate immune response [108]. Mitochondrially originated danger signals from damaged hepatocytes directly activate HSCs and drive the progression of liver fibrosis [68]. Nucleotide-binding oligomerization domain-like receptors (NLRP3) are a fundamental component of the inflammasome and the downstream targets of DAMPs [59,107]. Therefore, DAMPs exhibit a pivotal role in fibrogenesis and inflammation [4,5,107]. During hepatic fibrogenesis, hepatocytes alter their gene expression and secretion profile [2,4,5]. In addition, certain hepatocyte-derived molecules, including Notch, osteopontin, TGF-β, NADH oxidase 4, and Indian Hedgehog, have fibrogenic properties [109]. Moreover, damaged hepatocytes secrete exosomes that, inter alia, encapsulate microRNAs that promote HSC activation [2]. It is generally accepted that inflammation is a fundamental component in the process of liver fibrogenesis, while factors secreted from damaged hepatocytes alone cannot directly activate HSCs [2,68,107]. Ballooned hepatocytes display profibrogenic features and promote HSC activation by producing sonic hedgehog [2]. Hepatocytic cholesterol accumulation also results in HSC activation by stabilizing the transcriptional regulator TAZ in hepatocytes, which leads to the secretion of the profibrotic factor Indian Hedgehog [2,110]. Fatty acids, such as palmitic acid, promote the release of extracellular vesicles loaded with microRNAs (e.g., miR-128-3p). These microRNAs, when released from the extracellular vesicles upon their uptake by HSCs, inhibit the expression of peroxisome proliferator-activated receptor (PPAR)γ, which shifts quiescent HSCs to the activated HSC phenotype [108].

### 2.4. Liver Sinusoidal Endothelial Cells

Liver sinusoidal endothelial cells (LSECs) are unique endothelial cells that form the boundary of the hepatic sinusoids [110]. They are characterized by a lack of an organized basement membrane and the presence of open fenestrae that form a permeable barrier, which enables a direct connection between hepatocytes and access to oxygen, micronutrients, and macronutrients from the vessels [110,111,112]. In addition, they are permanently exposed to molecules translocated from the gut [110,111]. LSECs have an essential role in the maintenance of hepatic homeostasis, including regulation of the vascular tone, in inflammation and thrombosis, and in the control of hepatic immune responses [110]. In the healthy liver, differentiated LSECs are gatekeepers of fibrogenesis by maintaining HSCs in their quiescent state [111,112]. They play an important role in the secretion of vasoactive molecules, such as nitric oxide (NO) [113]. In addition, they act as regional antigen-presenting cells (APCs) that govern, in addition to hepatic DCs and KCs, intrahepatic immune responses by secreting cytokines and by activating or inhibiting immune cell signaling pathways [114,115]. LSECs actively participate in the clearance of antigens reaching the liver sinusoids and contribute to the perpetuation of the tolerogenic state [116,117]. They orchestrate the sinusoidal blood flow via their impact on HSCs and thus perpetuate a low portal pressure [111]. Furthermore, LSECs drive intrahepatic coagulation by several mechanisms, such as the production of pro- and anticoagulant factors, the recruitment and activation of neutrophils, and the crosstalk with platelets [116,117]. The fenestrated LSEC pheno type is orchestrated by two VEGF-dependent pathways, i.e., the VEGF-eNOS-soluble guan ylate cyclase (sGC)-cGMP pathway and a VEGF-dependent, NO-independent pathway [118].

LSECs play a critical role at all stages of chronic liver disease through four processes: sinusoid capillarization, angiogenesis, angiocrine signals, and vasoconstriction [111,114,115]. Chronic liver injury results in the capillarization of LSECs (a.k.a. dedifferentiation), which, as a result, lose their vasoprotective properties and take on pathological vasoconstrictive, proinflammatory, and prothrombotic properties [111,118]. Capillarization refers to a lack of fenestration and the development of a more continuous basal membrane [110,111,118]. In vitro studies with LSECs cocultured with HSCs revealed the role of capillarized LSECs on HSC activation; specifically, LSECs from normal rat liver prevented HSC activation and induced their reversion to a quiescent phenotype, whereas capillarized LSECs were not capable of inducing this phenotype [118,119]. A study in cirrhotic rats revealed that the administration of a sGC activator that induces the VEGF-eNOS-sGC-cGMP pathway restored LSEC fenestration, resulting in HSC quiescence and cirrhosis regression [120]. Oxidative stress related to NO signaling was implicated in LSEC dysfunction, resulting in fibrogenesis [117]. For example, reduced NO bioavailability has been observed in LSECs isolated from cirrhotic rat liver [117]. Capillarized LSECs secrete several cytokines and soluble molecules, influencing neighboring cells, promoting their dedifferentiation, and driving the development of chronic complications of liver disease, including portal hypertension [120]. Preclinical models of liver fibrosis have revealed the increased production of vaso constrictors by LSECs [121]. Activation of the cyclooxygenase 1 (COX1)-TXA2 pathway and endothelin 1 in rat chronic injury participate in sinusoidal contraction and exacerbate microvascular dysfunction [121,122]. Besides increasing vasoconstriction, capillarized LSECs have a defective eNOS-NO pathway; this entails portal hypertension and endothelial dysfunction [110]. Downregulation of eNOS and bioavailability have been shown in cirrhotic rat liver [123]. Gracia-Sancho et al. demonstrated that elevated hepatic oxidative stress in preclinical cirrhosis and impaired activity of the transcription factor KLF2 further contribute to diminish NO availability and exacerbate sinusoidal vasoconstriction [117]. KLF2 is a nuclear transcription factor sensitive to shear stress, enabling endothelial vasoprotection [110,118]. In experimental models of liver cirrhosis, liver endothelial KLF2 is upregulated as a compensatory mechanism driving the transcription of its vasoprotective target genes [124]. LSECs may also play a role in the pathogenesis of portal hypertension through a dysregulation of their antithrombotic capacity [125,126]. LSEC capillarization and the loss of the KLF2-dependent vasoprotective pathways promote the recruitment and activation of platelets (inducing microthrombosis and fibrin deposition within the hepatic sinusoids), which results in hypoxia and sinusoidal hypertension [110,125,126].

LSECs can maintain HSCs as quiescent, as long as they are differentiated [119,120]. VEGF contributes to the maintenance of LSEC differentiation [119]. There is a close relationship between liver fibrosis and angiogenesis in that fibrosis fosters angiogenesis and, in turn, liver angiogenesis exacerbates liver fibrosis [127,128]. In addition to LSECs, endothelial progenitor cells (EPCs), i.e., bone marrow-derived endothelial cells, also contribute to intrahepatic angiogenesis [129,130]. LSECs also orchestrate fibrosis through secreting angiocrine signals, paracrine molecules produced by endothelial cells that maintain organ homeostasis and regulate organ regeneration and tumor growth [131]. A recent study revealed that LSECs produce distinct angiocrine signals that maintain the balance between liver regeneration and fibrosis. Following acute liver injury, activation of the CXCR7-Id1 pathway in LSECs triggers the expression of hepatotropic angiocrine factors that drive liver regeneration. Con versely, chronic liver injury leads to persistent FGFR1 activation in LSECs, which inactivates the CXCR7-Id1 pathway and provides a CXCR4-driven profibrogenic angiocrine response, thereby provoking liver fibrosis [131]. Importantly, endothelial dysfunction occurs early in chronic liver disease, even before the onset of fibrosis and inflammation [131].

**Figure 2:**
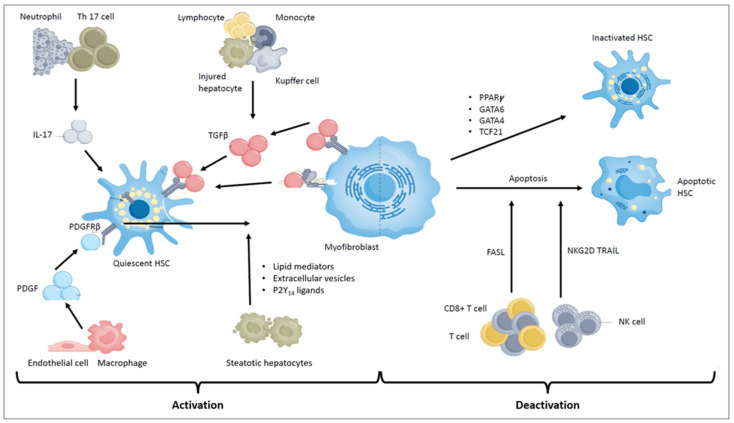
Molecular pathways and cellular interactions involved in HSC activation and deactivation. Activated HSCs are the main effector cells during hepatic fibrosis. In the healthy liver, they metabolize and store retinoids. Upon activation by fibrogenic stimuli, quiescent HSCs transdifferentiate into myofibroblasts, lose their vitamin A, upregulate α-smooth muscle actin (αSMA), and produce collagen I. Various factors, including immune cell-derived fibrogenic molecules, growth factors, and lipopolysaccharide, as well as profibrotic lipid mediators such as lysophosphatidylinositol and lysophosphatidic acid, induce HSC activation in the course of chronic liver disease. TGF-β is the most HSC potent activator, which is produced by infiltrating lymphocytes and monocytes, Kupffer cells (KCs), and damaged hepatocytes. IL-17, produced by neutrophils and Th17 cells, sensitizes HSCs to TGF-β by upregulating TGF-β receptor II (TGF-βRII). In addition, platelet-derived growth factor (PDGF), which is produced by endothelial cells and macrophages, further promotes HSC activation. During fibrosis resolution, HSCs either die or revert to an inactive state by upregulating transcription factors such as peroxisome proliferator-activated receptor-γ (PPARγ), GATA-binding factor 4 (GATA4), GATA6, and transcription factor 21 (TCF21). NK and CD8^+^ T cells can eliminate activated HSCs by inducing apoptosis (Further abbreviations: GM-CSF, granulocyte/macrophage colony-stimulating factor; HH, hedgehog ligands; IHH, Indian Hedgehog; LPA, lysophosphatidic acid; LPI, lysophosphatidylinositol; LPS, lipopolysaccharide; miRNA, microRNA; MSR1, macrophage scavenger receptor 1; NF-κB, nuclear factor κ-light chain-enhancer of activated B cells; OPN, osteopontin; oxLDL, oxidized low-density lipoprotein; ROS, reactive oxygen species; S1P, sphingosine-1-phosphate; SHH, sonic hedgehog; TLR4, Toll-like receptor 4). Modified from reference [5].

Figure 2 summarizes the most important molecular pathways and cellular interactions related to the activation and deactivation of HSCs detailed above. Further influencing factors are described subsequent to this figure.

### 2.5. Portal Fibroblasts

In the healthy liver, portal fibroblasts represent a small population of parenchymal cells that surround the portal venules and bile ducts, where they support the maintenance of the portal tracts’ architecture [4]. Portal fibroblasts were initially defined as periductal fibroblasts and periportal mesenchymal cells and were implicated in the pathogenesis of cholestatic liver injury in experimental models and patients alike [45]. TGF-β plays a key role in activating portal fibroblasts that produce ECM proteins in chronic liver disease [46]. While these cells are found in virtually every tissue, their molecular signatures differ between organs [45,46]. Single-cell technologies using mesenchyme-labeling PDGFβ production provided the transcriptomic and spatial in-depth characterization of a distinct subpopulation of portal fibroblasts both in healthy and damaged livers [45,46]. They become activated during the wound-healing process in response to cytokines and growth factors, including TGF-β1, IL-1-β, IL-6, or PDGF, and differentiate into myofibroblasts [1,46]. In activated portal fibroblasts, the crosstalk between mesothelin (MSLN), musin 16 (MUC16), and THY1 surface receptors orchestrates TGF-β1-TGF-βR1-SMAD2/3-promoted fibrogenic responses and proliferation via MSLN-AKT-FGFR1-dependent signaling [60]. Therefore, MSLN (a glycosyl phosphatidyl inositol-anchored membrane protein) and its ligand MUC16 (human analog ligand CA125) can obviously assume significant roles in the bio logy of activated portal fibroblasts [4,60]. Upon cholestasis, taurocholic acid promotes COL1A1 production in activated portal fibroblasts [40,43]. In addition, IL-25-triggered activated portal fibroblasts produce IL-13, which promotes the release of CTGF and HSC activation [43]. This finding may explain why the gene expression of cholestasis-activated HSCs is more similar to that of activated portal fibroblasts than that of activated HSCs in response to toxic injury. Consequently, activated portal fibroblasts might serve as a novel target for antifibrotic therapies [4,40].

### 2.6. Other Immune Cells in the Pathogenesis of Liver Fibrosis

Immune cells, such as T lymphocytes, macrophages, DCs, granulocytes, and mast cells, play important roles in the development and progression of fibrosis [132]. They produce many mediators driving inflammation and fibrogenesis, as well as the activation of fibroblasts and T lymphocytes, such as CD4^+^ and CD8^+^ T cells [132,133]. In patients with chronic liver disease, the number of effector CD8^+^ T cells increases [132]. Innate lymphoid cells (ILCs) constitute a subset of innate immune cells with lymphoid phenotypes; however, they do not express rearranged antigen receptors [132,133,134]. ILCs are classified into three groups based on the production of specific transcription factors, cell surface markers, and signature cytokines [134]. Group-1 ILCs (a.k.a. ILC1s) consist of IFNγ-producing and T-beta-dependent ILCs, group-2 ILCs (a.k.a. ILC2s) cover a subset of cells expressing type 2 cytokines such as IL-5 and IL-13, and group-3 ILCs (a.k.a. ILC3s) produce IL-17 and IL-22 [133,134]. Recently, this classification has been revised, and two novel members have been added to the ILC family, which are conventional NK (NK) cells and lymphoid tissue-inducer cells [134]. ILCs, particularly ILC1s and ILC3s, are involved in liver fibrogenesis [82,83]. Recent studies revealed that ILC1s are recruited in adipose tissue of type 2 diabetes patients, inducing fibrogenesis via the TGF-β signaling pathway [132,133]. ILC3s expressing IL-17 are increased in CCl_4_-induced cirrhotic mice [133,134]. The adoptive transfer of ILC3s into ILC-depleted mice led to ECM accumulation and induced liver fibrosis. These findings indicated a profibrogenic role of ILC3s in the progression of liver fibrosis [134,135]. Clinical and experimental studies have demonstrated that cNK cells are critically involved in the immune pathogenesis of chronic liver disease (CLD), and they display antifibrotic activity through the induction of apoptosis and/or killing of activated HSCs [132,133,135]. The antifibrotic activity of NK cells is associated with the surface expression of activating NK cell receptors, including NKG2D, NKp46, and NKp30, which recognize specific molecules produced by activated HSCs [134,135,136]. Forkel et al. revealed a link between the severity of liver fibrosis and the proportion of intrahepatic ILC2s, which may express IL-13 and exhibit profibrotic activity [137].

Myeloid DCs (in the DC-specific literature differentiating between three principal DC subclasses, this one is dubbed “classical” or “conventional”) assume a critical role in the development of liver fibrosis [138]. This is due to the fact that DCs serve as key professional APCs that regulate and orchestrate innate and adaptive immune responses in infections, chronic inflammatory diseases, cancer, autoimmunity, and, conversely, the induction of immune tolerance [132,138]. CCl_4_-induced liver fibrosis is more pronounced in HBV-transgenic mice as opposed to wild-type mice [139]. The depletion of NK cells and NKT cells or the blockade of CD1d inhibit α-SMA expression in the liver [139]. The number of mucosal-associated invariant T cells (MAIT cells) decreases in cirrhotic patients [140]. MAIT cells can promote HSC proliferation and the production of collagen and proinflammatory cytokines in vitro [140]. Increasing evidence suggests that adaptive immune cells, including Th1, Th2, and Th17 cells, also play important roles in liver fibrogenesis [141]. Novel studies have revealed that Tregs are relevant in the development of liver fibrosis, and it has been shown that signaling via the mammalian target of rapamycin (mTOR) is involved in orchestrating the protective function of Tregs [132,141]. Finally, B cells also promote the development and progression of liver fibrosis [132,141]. It has thus become increasingly evident that most immune cell populations and subpopulations normally participating in the realization of innate and adaptive immune responses are recruited in the context of liver fibrosis. While this certainly is, in part, due to fibrosis-specific activation processes, it can be reasonably assumed that this inflationary development is also a result of the excessive amount of soluble and cell membrane signals expressed in the progression of fibrosis. If so, any therapeutic efforts towards downregulating the detrimental immunological processes involved in liver fibrogenesis will be increasingly difficult the longer this process continues.

### 2.7. Metabolic Reprogramming of HSCs in Liver Fibrogenesis

HSCs regulate their energy expenditure to perpetuate their distinct functions during the development and progression of liver fibrosis [142]. Recent studies revealed that cells involved in the development and regression of liver fibrosis undergo metabolic reprogramming to meet their energy requirements [141,142,143]. The transition of quiescent to activated HSCs requires a high energy demand and therefore a reprogramming of the cellular meta bolic pathways [2,118]. This prominently includes significant changes in carbohydrate cata bolism, such as the upregulation of glycolysis, for the energy supply during the cells’ transition to myofibroblasts [142]. HSCs transform glucose to lactate to avoid oxidative phosphorylation (aerobic glycolysis), which has a weaker effect than oxidative phosphorylation in generating ATP [1,2,142]. Lactate reduces the extracellular pH and induces TGF-β1 activation [1]. The combination of low extracellular pH with an increased lactic acid level promotes myofibroblast transformation through the activation of TGF-β1 [1,144,145,146]. In the course of glycolysis, pyruvate is transformed to acetyl-CoA within the mitochondria [146]. Recent data documented that lactate is a significant metabolite in the activation and perpetuation phase of HSCs, and HSC activation requires more metabolic demands than ATP generation only [1,142]. Activated HSCs require high levels of aerobic glycolysis for the transition into the myofibroblast phenotype. Oxidative phosphorylation is one of the significant energy sources of activated HSCs, an indication of which is the increased number and activity of mitochondria [1,142]. This effect can be explained by the fact that these cells depend on both oxidative phosphorylation-generated ATP and mitochondria-derived ROS [142]. Excessive ROS production activates TGF-β signaling and stimulates inflammatory cells that, as detailed further above, contribute to hepatic fibrogenesis [1,142].

Preclinical trials using immortalized human-activated HSCs or primary murine HSCs, respectively, demonstrated an increase in enzymes that process glucose intracellularly after activation, including hexokinase 2 (HK2), fructose-2,6-biphosphatase-3 (PFKFB3), and pyru vate kinase (PK) [1,58,142]. HSCs upregulate their glycolytic pathway [1]. In addition, activated HSCs downregulate the expression of the proteins involved in gluconeogenesis, including phosphoenolpyruvate carboxykinase-1 (PCK1) and fructose bisphosphatase-1 (FBP1), and remove the central carbon metabolites from the TCA cycle to facilitate lactate accumulation [1,43]. However, these findings require being validated under physiological conditions, as they stem from in vitro experiments using primary or immortalized HSCs [1,4,53,58]. The metabolic reprogramming of HSCs and the enzymes involved in aerobic glycolysis are regulated by the activation of the Hedgehog pathway through the expression hypoxia-inducible factor 1-α (HIF-1α) together with TGF-β1 [43]. In addition, glutaminyl and protein metabolism are also upregulated by some enzymes, such as glutaminase 1 (GLS-1) [142]. During activation, HSCs have to master a significant bioenergetic challenge to fuel all their secretory functions, and their protein metabolism is reprogrammed as well. Du et al. showed the expression of genes involved in protein metabolism during carbohydrate metabolism [145]. They also reported that ~38% of genes differently expressed by quiescent HSCs and activated HSCs are involved in protein metabolism [75]. Glutaminolysis is the transformation of glutamine into α-ketoglutarate, which is usually identified in cancer cells and enables the generation of ATP needed for cell anabolism [75,142,145,146]. As previously mentioned, the utilization of vitamin A depots for the catabolism of fatty acids to enable an energy supply is one of the hallmarks of HSC activation [1,2,4,53,58]. During this process, LRAT expression is substantially reduced in activated HSCs, which causes a decrease in vitamin A storage and the progression of fibrosis [142,145,146,147,148]. 

The metabolism of lipid droplets during HSC activation generates fatty acids for β-oxi dation [43,147,148,149]. HSC activation is also promoted by transcriptional drivers of fatty acid content, including PPARγ and sterol regulatory-element-binding protein-1 (SREBP-1c). Increased glycolysis and glutaminolysis, together with enhanced fatty acid oxidation, have been considered to be relevant drivers of fibroblast activation [150]. Activated HSCs have been subdivided into three classes based on their expression profiles, being proregenerative (as characterized by increased growth factor expressions), antiregenerative, and a mixed phenotype [142]. During activation, the genes involved in retinol catabolism, including retinyl ester hydrolase (REH), are upregulated, while the enzymes involved in retinol esterification, including LRAT, are downregulated [142]. As a result, lipid droplets are lost and metabolized to activate the β-oxidation pathway. The enzymes involved in lipid metabolism, including the liver X receptors (LXRs), are upregulated, while adipogenic regulators, including sterol regulatory element-binding protein 1 (SEREB-1c), are downregulated [142]. Activated HSCs have been shown to increase the rate of aerobic glycolysis and related enzymes while decreasing the enzymes involved in gluconeogenesis [151]. Basal levels of glycolysis and mitochondrial respiration are substantially higher in activated rat HSCs when compared to quiescent HSCs [151]. This finding was associated with extensive mitochondrial fusion in rat and human-activated HSCs, which evolved without changes in the mitochondrial DNA content and electron transport chain (ETC) components [151]. ROS accumulates intracellularly and in the oxidative phosphorylation pathway, while the TCA pathway is downregulated [151]. Experimental trials detected that RNA-binding proteins such as cytoplasmic polyadenylation element binding protein 4 (CPEB4), human antigen R (HuR), and tristetraprolin (TTP) are critical regulators in HSCs [142].

### 2.8. Metabolic Regulation of Liver Fibrosis

Autophagy is a stress response mechanism that involves the degradation of cellular components and organelles through a lysosome-dependent pathway to generate energy and nutrients; this mechanism has been shown to also play a critical role in HSC activation and fibrogenesis [2]. Autophagy is required to perpetuate an activated phenotype in HSCs [2]. In mice with autophagy-defective HSCs, CCl_4_-induced liver injury leads to the cessation of ECM deposition and development of fibrosis [2,5]. Several profibrogenic and proinflammatory molecules, such as TGF-β and lipopolysaccharide, upregulate autophagy in HSCs [152,153]. TGF-β-induced autophagy displays a role in HSC activation through the c-jun *N*-terminal kinase and extracellular signal-regulated kinase signaling pathways [152]. The lipopolysaccharide-induced upregulation of autophagy modulates the suppression of the TGF-β pseudo-receptor BAMBI [153]. In addition, macrophage-derived PGE_2_ can drive HSC activation and fibrosis through promoting autophagy [154]. In a mouse model of diet-induced fatty liver disease, M2 macrophages were found to induce HSC autophagy by expressing PGE_2_, ultimately fostering HSC activation, ECM production, and the develop ment of fibrosis [154]. Antagonization of the PGE/EP4 pathway suppresses HSC autophagy and regresses liver fibrosis [154].

Autophagy drives HSC activation by fatty acids formed as a result of the cleavage of retinyl esters within lipid droplets [109,155]. In experimental mouse models of CCl_4_-induced liver fibrosis, autophagy-related protein 7 (ATG7), fibrogenesis, and ECM accumulation were found to be remarkably reduced, but this finding could not be replicated in cultured HSCs by adding exogenous fatty acids [156]. Endoplasmic reticulum (ER) stress signals promote gene expression in HSCs by inducing autophagy and HSC activation [118,157,158]. Targeted lentiviral delivery of the GRP78 protein, an ER stress protein, can inhibit fibrogenesis following CCl_4_ treatment. Inhibition of inositol-requiring enzyme 1 (IRE1) in HSCs decreases both their activation and autophagic activity and leads to reduced fibrogenic activity [118,157,158]. Ectopic overexpression of X-box binding protein (XBP1), a transcription factor downstream of IRE1, promotes type 1 collagen production in HSCs, which is suppressed by the knockdown of ATG7 [2,118,158]. Another signaling pathway activated by ER stress is PKR-like endoplasmic reticulum kinase (PERK), which stimulates HSC activation. ER stress in HSCs induces hepatic fibrosis by dysregulating miR-18, which is regulated by the activation of PERK and the destabilization of heterogeneous nuclear ribonucleoprotein A1 (HNRNPA1) [118,158]. Heat shock protein 47 (HSP47) is a collagen-specific molecular chaperone that assumes critical roles in the expression of type 1 collagen [2,4,5,155]. In HSP47-depleted HSCs, immature type 1 procollagen accumulates [1,2,4,5,155]. When autophagy is inhibited in these cells, ER stress and apoptosis are increased compared to wild-type HSCs [4]. JANK signaling, which merges downstream of ER stress, was associated with hepatocyte damage, NAFLD, and hepatic fibrosis [1,4]. JNK1 signaling in HSCs strongly promotes liver fibrosis; however, JNK1 signaling does not affect hepatocytes in a similar manner [1,4]. An experimental trial in mice has indicated that liver fibrosis is substantially diminished in JNK1-deficient mice compared to wild-type mice and in those with hepatocyte-specific JNK1 deficiency in response to CCl_4_ or BDL [159].

### 2.9. Epigenetic Regulation of HSCs

Recent data indicate that the transition of quiescent HSCs to activated HSCs requires an epigenetic mechanism to silence adipogenic differentiation factors and enhance the de novo expression of genes related to novel phenotypes [2,4,5]. There are three fundamental mechanisms of epigenetic regulation, i.e., noncoding RNA (ncRNA) expression, DNA methylation, and histone modification [160]. Among several endogenous factors regulating gene expression, certain miRNAs are essential [161]. In chronic liver disease, some signaling pathways are promoted by inflammatory molecules triggering liver fibrosis; these signaling pathways are orchestrated by miRNAs [160,161,162]. MiRNAs are single-stranded noncoding RNAs containing about 18–25 nucleotides and drive post-transcriptional gene expression through altering mRNA degradation [163]; they display either profibrotic or antifibrotic effects [162,163]. Experimental studies identified several profibrogenic miRNAs, including miR-21, miR-221, miR-222, and miR-27 [162,163]. Conversely, miR-214, miR-378a, miR148a, miR-133a, and miR-195 display antifibrotic effects [162,163]. The miR-15 family induces cell proliferation and promotes apoptosis, while the miR-29 family orchestrates the accumulation of the ECM and promotes apoptosis by modulating the P13/AKT signaling pathway [162]. MiR-29 family members promote various signaling pathways, such as TGF-β, NF-κB, and P13K/AKT, promoting liver fibrosis [144,162]. The interaction between miR-29b and the TGF-β/Smad3 signaling pathway arises in activated HSCs. Smad3 negatively regulates miR-29b expression, which directly drives the TGF-β/Smad3 pathway and induces liver fibrosis [164]. Additionally, miR-34 stimulates HSCs and promotes liver fibrosis in rats by inducing acyl-CoA synthetase, which exhibits a critical role in hepatic lipid metabolism [144,162,164]. Furthermore, a recent trial revealed that a lack of miR-21 in NASH-associated chronic liver disease leads to decreased steatosis, inflammation, and lipoapoptosis, resulting in decreased fibrosis [142]. Myocardin-related transcription factor A (MRTF-A) drives profibrogenic transcription by accumulating a histone methyltransferase complex in the promoters of fibrogenic genes [1,4,5]. MRTF-A-deficient mice show resistance to CCl_4_-induced fibrosis [1,109]. Long noncoding RNAs (lncRNAs) are a class of transcripts with >200 nucleotides in length that act as regulatory RNAs [165]. They extensively modulate gene expression at the transcriptional, post-transcriptional, and epigenetic levels by interacting with proteins, DNA, or mRNAs [165]. Studies increasingly reveal lncRNAs as relevant drivers of fibrogenesis [165]. In multidrug resistance 2 knockout (Mdr2^-/-^) mice, cholangiocyte-derived exosomal H19 is transferred to adjacent hepatocytes and HSCs, which propels the progression of cholestatic liver fibrosis by modulating bile acid accumulation in hepatocytes and promotes the activation and proliferation of HSCs [165,166]. When interacting with the enhancer of zeste homolog (EZH2), lncRNAs can orchestrate the progression of fibrosis [165].

As mentioned above, activated HSCs alter their gene expression profile and transdifferentiate into a profibrogenic myofibroblast phenotype. The existence of various transcription factors was suggested to orchestrate this process, but suitable models on the reprogramming of the myofibroblast epigenome are still missing [167]. Epigenetic mechanisms may participate in these signature transcriptional processes during HSC activation [167,168]. DNA CpG methylation exhibits a role in the global DNA alteration during HSC activation [168]. Methylated CpG motifs within the promoter of PPARγ accumulate methyl-CpG-binding protein 2 that subsequently promotes H3K9 methylation and accumulates transcriptional repressor chromobox protein homolog 5, which inhibits PPARγ production and drives HSC activation [169]. Page et al. investigated whether HSC activation may be accompanied by DNA methylation remodeling; they compared the global changes of 5-metylcytosine and 5-hydroximethycytosine during HSC activation [168]. Methylation of CpG plays an essential role in gene silencing, and 5-mC may be additionally oxidized to secrete 5-hmC, which is generally detected in transcriptionally active genes [167]. The researchers also investigated the production of DNA methyltransferases (DNMT1, 3a, and 3b) orchestrating the annotation of 5-mC and methylcytosine dioxygenase (TET) that oxidize 5-mC to 5-hmC [168]. In this study, the production of TET proteins was found to be uniformly reduced in both animal and human models, with a concurrent decrease in the global levels of 5-hmC in fibrotic livers compared to healthy controls [168]. In contrast, the expression of DNMT proteins tended to increase in fibrotic liver, but the increase in the DNMT level did not change the global amount of 5-mC, which may be due to changes in DNMT activity [168]. The researchers suggest that DNA 5-mC/5-hcM is a pivotal step in HSC activation and fibrogenesis [168]. Changes in DNA methylation during HSC activation may add novel perspectives to the molecular mechanisms underlying fibrogenesis and may lead to the discovery of novel drugs and biomarkers that might serve to prevent or delay the progression of liver fibrosis.

Liver fibrosis is further associated with alterations in DNA methylation patterns and the expression of epigenetic enzymes involved in local fibrogenesis [161,162]. A genome-wide study of DNA methylation patterns in CCl_4_ mouse liver tissues revealed the hypomethylation of fibrosis-related genes before the onset of liver fibrosis [170]. Furthermore, on the third day of culture, a ~60% loss of original DNA methylation levels was detected in rat HSCs, a condition that triggered fibrogenic activity [171]. Although the transition of HSCs to myofibroblasts is associated with a general loss of DNA methylation, the presence of gene-specific DNA hypomethylation and hypermethylation has been documented in genome-wide DNA methylation studies [171,172]. For instance, upregulation of profibrogenic genes such as Actg2, Loxl1, Loxl2, and Col4A1/2 is associated with a decrease in promoter methylation levels in activated HSCs [173]. Conversely, downregulation of the Smad7 and Pten genes is associated with DNA hypermethylation [174]. DNA methylation is orchestrated upon the attachment of methyl-binding proteins, which subsequently accumulate the transcriptional repressor complex [175]. The expression of MeCP2, a methyl-binding protein, is promoted during the transition of HSCs to myofibroblasts [176]. MeCP2-deficient mice displayed attenuation of the expression of fibrogenic markers such as collagen-1, TIMP-1, and α-SMA and regression of liver fibrosis after CCl_4_ treatment [177]. PPARγ must be silenced for HSCs to be activated and to transdifferentiate into the myofibroblast phenotype [178]. MeCP2 silences PPARγ via different mechanisms [178]. Specifically, MeCP2 represses PPARγ expression either by binding to methyl-CpG residues in the PPARγ promoter or by enhancing EZH2 expression [178]. CpG methylation is driven through DNA methyltransferases (DNMTs) [42,57]. DNMT-mediated hypermethylation of phosphatase and tensin homolog (PTEN) initiates the activation of HSCs [39,49]. Many studies have revealed the key role of histone methylation in liver fibrosis. The MeCP2-mediated transdifferentiation of HSCs is, in part, regulated by the two distinct histone methyltransferases EZH2 and ASH1 [179]. Their expression is promoted during HSC activation, leading to fostering H3K27me3 and H3K4me modifications, respectively [162]. Overexpression of EZH2 promotes the expression of fibronectin, α-SMA, and collagen 1α1 in HSCs [180]. Studies in in vitro and in vivo models of CCl_4_ and BDL-induced liver fibrosis have demonstrated that EZH2 inhibitors such as 3-deazaneplanocin A (DZNep) and GSK-503 have antifibrotic properties [181]. Methyl-CpG-binding protein and histone-lysine *N*-methyltransferase enhancer of zest homolog 2 (EZH2) regulate epigenetic signaling by repressing PPARγ [182]. The Wnt/β-catenin signaling pathway activates HSCs by increasing the MeCP2 protein levels, which, in turn, represses PPARγ [183].

**Figure 3:**
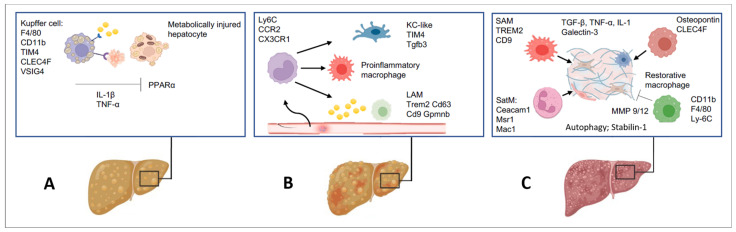
The path towards liver fibrosis: Kupffer cell activation and macrophage recruitment in the chronic inflammatory microenvironment of the diseased liver. (**A**) Ingestion of fat-laden apoptotic hepatocytes and free cholesterol activates KCs by promoting the production of proinflammatory mediators. (**B**) The liver’s chronic inflammatory microenvironment recruits monocytes from the circulation, which, due to local proinflammatory signaling, differentiate into monocyte-derived KC-like inflammatory, as well as lipid-associated, macrophages. (**C**) Macrophage populations are the major contributors in shaping both profibrotic and antifibrotic drivers within the fibrotic niche. Relevant phenotypic markers of the macrophage populations detected in mouse models are indicated in the figure (Abbreviations: CEACAM1, carcinoembryonic antigen-related cell adhesion molecule 1; CLEC4F, C-type lectin domain family 4 member F; LAM, lipid-associated macrophage; Mac1, macrophage-1 antigen; Mar1, macrophage scavenger receptor1; MMP, matrix metalloproteinase; SAM, scar-associated macrophages; SatM, segregated nucleus-containing atypical monocytes; TGF-β, transforming growth factor-β; TNF-α, tumor necrosis factor-α; VSIG4, V-set and immunoglobulin domain containing 4).

Before discussing the mechanisms of regression of liver fibrosis, we present a sketch of the changes in the hepatic macrophage populations on the path to liver fibrosis (Figure 3).

## 3. Mechanisms Driving the Regression of Liver Fibrosis

Single-cell technologies have led to an unprecedented comprehensive understanding of the key mechanisms underlying the reversibility of liver fibrosis, resulting in the identification of new antifibrotic targets [95]. The interaction between damaged hepatocytes, inflammatory cells, and activated HSCs results in liver fibrosis [1,2,3,13,168]. As such, eliminating the cause of chronic liver injury is the main goal of antifibrotic treatment [2,4,5]. Compared to chronic injury in other organs, the progression of hepatic fibrosis is extremely slow, and the development of liver cirrhosis takes several decades [4,5]. The slow progression of liver fibrosis is explained by the liver’s extraordinary regeneration capacity [4,5,59]. Recent data from experimental and clinical studies have proven that liver fibrosis is a dynamic and reversible process [2,4,5,13,118]. In some chronic liver diseases, such as hepatitis B, hepatitis C, autoimmune hepatitis, and NASH, sequential liver biopsies revealed that liver fibrosis regressed or disappeared upon cessation of the underlying cause [4,5]. In addition, bariatric surgery and weight loss have been documented to inhibit insulin resistance and counteract the development or progression of the metabolic syndrome, as well as lead to the resolution of liver fibrosis in some NASH patients [2,4]. In experimental models of liver fibrosis caused by CCl_4_, ALD, and BDL, liver fibrosis regresses when the underlying cause is removed [184]. The resolution of liver fibrosis is associated with a decrease in the levels of some cytokines, such as IL-6, IL-1-β, TNF, and TGF-β, in the liver [184]. TGF-β signaling is a key driver of fibrogenesis; when this signaling is blocked, the number of activated HSCs declines rapidly, ECM production stops, and MMP production is upregulated [2,3,184].

## 4. The Fate of Fibrogenic Myofibroblasts

### 4.1. HSC Apoptosis

Cellular biological processes, including apoptosis, senescence, and reversion to quiescent HSCs, lead to the deactivation of activated HSCs [2,5,118]. So far, there are only limited data on the biology of HSCs in the course of liver fibrosis regression. Its underlying basic mechanisms are usually investigated in experimental mouse models [3,183,184,185]. Preclinical studies on myofibroblast survival have shown that the apoptosis of activated HSCs is a key pathway of liver fibrosis regression [2,3,186,187,188]. From a cellular regulatory perspective, apoptosis is a form of programmed cell death that drives the disbalance between HSC proliferation and death during hepatic fibrogenesis [2,4,5,118,187]. This cellular process can promote the decrease in the number of myofibroblasts; however, this is insufficient to reconstitute the liver tissue’s integrity [186]. The apoptosis of activated HSCs is regulated by an intricate crosstalk between proapoptotic and prosurvival signals in activated HSCs in the healing liver, in which ~50% the of activated HSCs are subject to apoptosis [4,5,118]. Given the significant plasticity of HSCs and the ability of quiescent HSCs to differentiate into myofibroblasts, activated HSCs may also differentiate into quiescent HSCs [118,184]. Once toxic liver damage is halted, activated HSCs evade apoptosis, reduce their expression of fibrogenic genes, and differentiate into quiescent HSC states [189,190,191,192,193].

Two fundamental apoptosis signaling pathways have been identified: the extrinsic pathway and the intrinsic pathway. The intrinsic pathway, i.e., the mitochondrial apoptotic pathway, is activated by mitochondrial outer membrane permeabilization (MOMP) in the presence of intracellular stimuli [194]. MOMP, which is orchestrated by members of the BCL-2 family, triggers apoptosome formation, which, in turn, leads to cell death [194]. In addition to activating the intrinsic apoptosis pathway, MOMP plays a critical role in mitophagy, which is the selective intralysosomal degradation of mitochondria by means of a specific autophagic pathway [194]. Mitophagy is orchestrated by distinct dynamics, and the PINK1/Parkin pathway is thought to be the most relevant regulatory path [4,5,186,194]. So far, only a limited number of studies on the process of mitophagy in HSCs and its effect on liver fibrosis have been published. In addition to being the major regulator of apoptosis, the BCL-2 family proteins exhibit other functions, including mitophagy [186,194]. BCL-B is a recently identified member of the BCL-2 family that has a proapoptotic effect on some cancer cells [186]. However, the role of BCL-B on activated HSC apoptosis and mitophagy is largely unknown to date [2,4,5,194]. Ding et al. demonstrated that mitophagy is fostered and associated with increased apoptosis in HSCs during the resolution of liver fibrosis. The inhibition of mitophagy mitigates apoptosis in HSCs and induces hepatic fibrosis in mice. In contrast, the activation of mitophagy promotes apoptosis in activated HSCs. In addition, the knockdown of BCL-B increases apoptosis and mitophagy, while BCL-B overexpression results in the opposite effect [186].

Several mechanisms can play important roles in the apoptosis of activated HSCs, including activation of death receptor-mediated pathways (FAS or TRAIL), caspases 3 and 8, upregulation of proapoptotic proteins such as p53 and BAX, and activation of liver-related NK and NKT cells [186,187,188]. TNF-α-related apoptosis-inducing ligand/Apo-2-ligand (TRAIL) is a type 2 transmembrane protein in the TNF-α superfamily that can promote apoptosis in cells expressing TRAIL receptor 1 (TRAIL-R1), also known as death receptor 4 (DR4) [186,187,194]. TRAIL has sequence homology with TNF and FasL [195]. It is strongly expressed by cells of the innate immune system, particularly NK cells, and can be proteolytically cleaved from the cell surface and released in soluble form [187]. Of five TRAIL receptors currently documented in humans, TRAIL-R1/DR4 and TRAIL-R2/DR5 trigger apo ptosis [188]. The HSC cell line LX2 overexpresses DR4 and DR5 and becomes sensitive to TRAIL-induced cell death. Despite various experimental studies, the role of TRAIL signaling in liver fibrogenesis remains to date largely unknown [187]. Moreover, an active agent for inducing apoptosis in activated HSCs with limited hepatotoxicity has not yet been developed. Previous in vitro studies suggested that activated HSCs overexpress DR5 and DR4 and acquire sensitivity to TRAIL-mediated apoptosis [188,196]. Gao et al. showed that NK cells could deplete activated HSCs in murine models of liver fibrosis via a TRAIL-mediated mechanism. These findings proved that TRAIL may be a potential proapoptotic agent to deplete activated HSCs in liver fibrosis and cirrhosis [195]. Oh et al. showed that the pharmacological depletion of activated murine HSCs in vivo can be achieved by employing recombinant human TRAIL [188]. They also documented the overexpression of DR4 and DR5 by hepatic myofibroblasts in vivo and suggested a mechanistic explanation for the sensitivity of activated HSCs to TRAIL-mediated apoptosis [188]. 

Proinflammatory signaling, such as via NF-kB activation, leads to the expression of profibrogenic genes and induces resistance to apoptosis [1,2,4,5]. On the other hand, many signals (e.g., FXR, CB2, and adiponectin) promote apoptosis in activated HSCs [1,5]. It has been estimated that ~50% of activated HSCs are eliminated by apoptosis during the regression liver fibrosis [196]. Using Cre-loxP-based genetic labeling of myofibroblasts, in Col1a1-GFP mice [i.e., transgenic mice expressing collagen aI (I) promoter/enhancer-driven green fluorescent protein (GFP)], the cellular fate of activated HSCs during the resolution of CCl_4_-induced fibrosis has been demonstrated [184,197,198]. Following apoptosis-driven HSC loss, the HSC population was found completely improved in their functional role towards the liver’s pericytes, as well as in their vitamin A storage capacity around day 7 after CCl_4_ cessation [197,198,199,200]. In the recovered liver, HSCs did not express activation markers one month after halting CCl_4_, and newly generated HSCs in the recovered liver exhibited all properties of a quiescent HSC phenotype that cannot be distinguished from quiescent HSCs isolated from treatment-naïve mice [199]. Almost half of the activated HSCs died after ceasing CCl_4_-dependent liver injury [198,199,200]. These observations demonstrate that the regression of liver fibrosis, at least in part, is a dynamic process due to HSC transformation in the healing liver [4,197,198,199,200].

### 4.2. HSC Senescence

While a considerable portion of activated HSCs dies and undergoes apoptosis shortly upon suspending CCl_4_-related liver injury, there may also be a critical role of senescence in the resolution of liver fibrosis [1,4,58]. However, although many studies have been performed, the fate of the senescent cells has not yet been determined due to the absence of unambiguous senescence markers [188]. Senescence is an irreversible mechanism of cell death contributing to the eradication of activated HSCs during fibrosis resolution [1,4,5,188]. Cellular senescence is a form of proliferation arrest characterized by the inhibition of E2F target genes and an exhaustion of the cells’ replicative properties. During this physiological process, proliferating cells enter a state of stable cell cycle arrest that prevents the cells from responding to mitogens [187,191,192]. In addition to apoptosis, senescence is another fate of activated HSCs during the liver fibrosis resolution [191]. Senescent-activated HSCs originate in the fibrotic liver, remain metabolically active, and display gene expression profiles consistent with the cell cycle exit [133,191]. Senescent HSCs produce several biologically active molecules that drive immune surveillance and inflammation and orchestrate tissue homeostasis, fibrosis, and fibrosis regression [4,133,191]. HSCs display a so-called “senescence-associated secretory pheno type”, which results from the downregulation of Dnase2 and TREX1 [191]. The ECM production in activated HSCs that become senescent is reduced so that fibrogenesis decreases [187,191]. Senescent HSCs cease to proliferate due to telomere shortening, chromatin modifications, DNA damage, oncogene activation, inactivation of tumor suppressor genes, and cellular stress [133,191]. 

Activated HSCs that are subject to replicative senescence display a less fibrogenic phenotype and usually undergo apoptosis [191]. Experimental and clinical studies have shown that liver myofibroblasts express senescence markers [2,3,4,5]. The senescence of myofibroblasts is driven via the p53 and p16 pathways [1,2,4,5]. Studies using cell-specific p53 knockout mice have demonstrated that the inhibition of cellular senescence in activated HSCs caused increased HSC proliferation and progression of liver fibrosis [191,201]. Senescence of activated HSCs is associated with telomere shortening [1,4,5]. Following the halting of CCl_4_ injury, p53 suppression causes the persistence of activated HSCs and stops liver fibrosis resolution [202]. These findings have demonstrated that senescence of activated HSCs is a relevant mechanism in the eradication of HSCs in the context of liver fibrosis resolution [190,201]. Additionally, in vitro studies investigating the characteristics of senescent HSCs have demonstrated that senescence leads to a decreased production of ECM proteins, increased synthesis of MMPs, downregulation of the BCL2 gene, and upregulation of immune surveillance-associated genes [192,201]. IL-22 promotes HSC senescence and enhances liver fibrosis degradation [1,2,4,5]. Senescent HSCs are found during the resolution of liver fibrosis, where they contribute to mitigating the progression of fibrosis. Therefore, induction of senescence in HSCs is a promising potential antifibrotic strategy [191,192]. The recruitment of immune cells involved in the removal of activated HSCs, such as NK cells, also contributes to this process [201]. Gene expression arrays suggest that, in the senescence-associated secretory HSC phenotype, GFAP expression declines [1,2,3]. In addition, the lower expression of genes related to cell migration, such as CXCR4/C-X-C motif chemokine receptor 4 and matrix metalloproteinase 13 (MMP13), and the lower expression of integrins have been identified during individual aging [1,2,3,4,5]. Furthermore, aging impairs HSC functions due to the lower expression of ECM-related genes and growth factors, including the hepatocyte growth factor (HGF) [3].

### 4.3. HSC Inactivation

The reversal of HSC activation was documented in rodent models of fibrosis resolution [1,2,4,5]. During fibrosis regression, activated HSCs are eliminated either via apoptosis or by reverting to a quiescent HSC phenotype, where they remain in a primed state and respond more rapidly to new fibrogenic stimuli [2,4,5]. Studies in transgenic mice have revealed that activated HSCs can revert to a quiescent state after eliminating the etiological factor of liver fibrosis [192,195]. Kisseleva et al. investigated HSC deactivation using transgenic mice with CCl_4_- or ethanol-induced liver fibrosis [184]. After long-term complete recovery, fluorescent quiescent HSCs have been observed in the liver [184]. Troger et al. studied the activation and deactivation of HSCs in a BAC-transgenic mouse using two complementary approaches, i.e., scPCR and genetic cell fate tracking [193]. They thus demonstrated that HSC activation markers, such as expression of the Col1a1 and TIMP1 genes, gradually and continuously decreased in almost all of the activated HSCs during the recovery period after CCl_4_ and thioacetamide treatment [193]. The authors suggested that HSCs are deactivated in the healing liver [193]. Similar findings have been recorded in transgenic mice l, in which 40% of HSCs reverted to a quiescent phenotype in the recovering liver [192]. Importantly, during the resolution of liver fibrosis, the location of HSCs shifts from the fibrotic septa to a perisinusoidal site [193]. The activation of liver-resident NK cells can contribute to the resolution of liver fibrosis by killing activated HSCs [193]. Additionally, NK and NKT cells are a major source of IFNγ that has a potent antifibrogenic effect and promotes HSC apoptosis [195]. Hepatic NKT cells can exhibit a dual, either protective or pathogenic, role in liver diseases. In the setting of liver injury in mice, hepatic CXCR6^+^ NKT cells promote the release of inflammatory cytokines such as IFNγ and IL-4 that drive liver fibrosis [195,203]. The upregulation of the antiapoptotic genes HSPA1A or HSPA1B affects the survival of inactivated HSCs [184]. Blocking TLR4 signaling in mice or reducing hepatic exposure to intestinal microorganisms improves liver fibrosis [81].

PPARγ is a key nuclear receptor maintaining the quiescent HSCs phenotype. At least in mice, it can also revert already activated HSCs to an inactivated quiescent-like phenotype [109,187]. Studies in cultured HSCs have comprehensively documented the role of PPARγ [195]. Recent studies have revealed that the overexpression of PPARγ in activated HSCs leads to their regression to the quiescent phenotype [2,4,5,187]. When cultured activated HSCs are treated with adipocyte differentiation or exposed to basement membrane-like ECM, they upregulate adipogenic transcription factors, which results in the morphological and biochemical regression of activated HSCs to quiescent cells [184]. Epigenetic factors such as the methyl-CpG-binding protein 2 (MeCP2) mediate silencing of the PPARγ-encoding gene or the transcriptional activation of profibrogenic genes via specific histone modification [184]. These experimental findings have recently been confirmed in vivo: HSC-specific PPARγ knockout mice are more sensitive to CCl_4_-induced hepatic fibrosis, and when the fibrogenic CCl_4_ agent is ceased, liver fibrosis in mice with specific PPARγ knockout resolves more slowly than in wild-type mice [195,198,203].

## 5. Potential Novel Therapeutic Targets for Treating Liver Fibrosis

While no drug has as yet been approved for the treatment of liver fibrosis, some novel treatment approaches have been shown to be effective in patients with chronic viral hepatitis that were successfully treated with antivirals [58,199]. Comprehensive information on the molecular and cellular mechanisms underlying fibrosis resolution is required to develop potential novel drugs [58,204,205]. Targeting ECM remodeling can be an effective approach [187]. A range of candidate proteases that degrade scar components or downregulate or neutralize their cellular sources have been identified, resulting in models of matrix degradation in the liver. Both HSCs and KCs have been considered to be sources of matrix metalloproteinases (MMPs) [53,58]. The activity of these enzymes is further orchestrated by inhibitory molecules referred to as tissue inhibitors of metalloproteinases (TIMPs) [74,187,198]. A key study published by Duffield et al. revealed macrophages as a source of MMPs by demonstrating that their depletion in mouse models influenced the level of fibrosis resolution [206]. Another study in mouse models of liver fibrosis demonstrated that the resolution is delayed by KC depletion and accelerated by an adoptive transfer of KCs from wild-type animals, compared to KCs from MMP9^–/–^ mice, suggesting that KC-derived MMP9 is essential in resolving fibrosis [207]. The second messenger cAMP drives fibrosis regression and could be a potential target to slow down fibrosis [205,208]. The Wnt/β-catenin signaling pathway plays a critical role in HSC activation; therefore, this pathway could also be a potential target for resolving fibrosis [209]. Farsenoid X receptor (FXR) is a key regulator of hepatic bile homeostasis, lipoprotein, and glucose metabolism, which has inhibitory effects on HSC activation [210,211]. Obeticholic acid, the first molecule to target FXR to be approved by the FDA in 2016 as a second-line treatment for primary biliary cholangitis, has been shown to improve fibrosis in NASH patients [211]. Apoptosis inhibitors such as the pan-caspase apoptosis inhibitor emricasan have been investigated to determine their antifibrotic roles [212]. KCs could be targeted by distinct approaches, including the reduction of circulating monocyte recruitment, the inhibition of KC activation, and the modulation of proinflammatory macrophage polarization [202]. 

Exosomes, as communication vesicles released by all cell species in both physiological and pathological conditions, may also be involved in the regression of liver fibrosis [213]. Quiescent HSCs secrete exosomes that reduce HSC activation, while regular hepatocytes produce vesicles that can reduce the expression of profibrogenic genes [213]. Exosomes released into the serum might be useful antifibrotic tools [214]. Additionally, stem cell transplantation, including MSCs, endothelial progenitors, and hematopoietic stem cells, is an effective means to repair fibrotic livers in experimental models; they stimulate hepatic proliferation, inhibit activated HSCs, increase MMP activity, and induce neovascularization [214,215]. The combination of the bacterial metabolites LPS and TLR4 plays a critical role in the development of hepatic fibrosis by activating intrahepatic fibrogenic cells. Therefore, the inhibition of TLR4-related intracellular signaling may be effective in reducing TLR4-mediated inflammation and potentially inhibit liver fibrosis [109]. Recently, Lin et al. suggested a therapeutic strategy by targeting leukocyte-derived chemotaxis 2/tyrosine kinase with immunoglobulin-like and epidermal growth factor-like domain (LECT2/Tie1) signaling with bevacizumab (anti-VEGF/VEGFR signaling) to reverse fibrosis [199,216,217].

## 6. Note Added in Proof

We include here a separate section with some additional aspects of liver fibrosis that our referees reasonably requested to be considered in this review article; these aspects could not have been inserted into the existing text body without disrupting its logical flow.

Chronic hepatitis B and chronic hepatitis C are the leading causes of liver cirrhosis, HCC, and liver-related mortality worldwide. While HCV can be completely eradicated by direct-acting antivirals (DAAs), a functional cure for HBV infection is not yet available, but viral replication can be effectively suppressed and controlled with antivirals such as entecavir and tenofovir. Still, better therapeutic strategies are currently being developed [218,219,220]. The regression of liver fibrosis in patients with HCV-related liver cirrhosis with a sustained virological response upon DAA treatment could be demonstrated by related pathological parameters and/or by measuring liver stiffness with transient elastography (FibroScan) [221,222]. Also, a recent study of 911 patients with HBV-related compensated cirrhosis who received antiviral treatment with entecavir or tenofovir showed a regression of liver fibrosis [i.e., improvement in the Fibrosis 4 Index (FIB-4) from the first year of treatment] and a lower risk of developing HCC [223].

## 7. Conclusions and Future Perspectives

Hepatic fibrosis is a major global healthcare burden. Long-term liver damage caused by any etiology results in fibrosis because of a dysfunctional wound-healing process with an abundant accumulation in the ECM. Experimental and clinical trials have demonstrated that liver fibrogenesis is a dynamic process involving both parenchymal and non-parenchymal liver cells, as well as infiltrating immune cells. After approximately four decades of continuous progress in basic, translational, and clinical studies, the mechanisms underlying the pathogenesis of liver fibrosis have been largely elucidated, as well as the key drivers of hepatic fibrosis, stimulating the increased accumulation of ECM compounds. Inflammation is closely associated with fibrosis; macrophages thus play critical a role in liver fibrogenesis, but since they also participate in the regression of fibrosis, these cells display typical Janus-faced characteristics due to their highly plastic nature. On a molecular basis, the fibrogenic response is orchestrated by various players engaging their cell surface receptors and triggering downstream signaling pathways, which promote the expression of ECM proteins. Compared to the long-term fibrosis-related damage observed in other organs, liver fibrosis progresses very slowly, and the development of liver cirrhosis takes decades.

There is no currently approved drug for the treatment of liver fibrosis. However, clinical and experimental studies have demonstrated that hepatic fibrosis regresses dramatically when an underlying etiologic trigger is eliminated. Perhaps this consistent observation indicates the direction in which effective therapeutic approaches will develop. Liver fibrosis resolution is an intricate and closely orchestrated event involving a range of cell species and molecular classes. Despite tremendous advances during the last decade in our understanding of the pathogenesis of liver fibrosis, many challenges still remain that currently prevent the translation of this knowledge into effective antifibrotic therapies. Liver fibrosis resolution is associated with increased collagenase activity, the activation of KCs that secrete MMPs, and ECM degradation. Senescence and apoptosis of activated HSCs play a substantial role in the resolution of liver fibrosis when eliminating the cell type responsible for triggering fibrotic scar development. Some myofibroblasts revert to an inactive phenotype corresponding to quiescent HSCs. The numbers of myofibroblasts derived from both HSCs and portal fibroblasts are reduced when liver fibrosis regresses. Although liver fibrosis resolution provides a promising approach to the treatment of chronic liver disease, additional trials are required to gain an even more comprehensive understanding of the underlying mechanisms of hepatic fibrogenesis in order to conceive novel effective drugs for this condition. Experimental models and ex vivo primary human tissue culture systems enabling a better translation of novel mechanisms from bench to bedside should be established. Fortunately, the discovery of antifibrotic mechanisms has meanwhile become an extremely attractive field of research.

## Abbreviations

αSMA	α-smooth muscle actin
APCs	antigen-presenting cells
ATP	adenosine triphosphate
BAMBI	BMP activin membrane-bound inhibitor
CEACAM1	carcinoembryonic antigen-related adhesion molecule 1
CLD	chronic liver disease
COL1A	collagen 1A
DAMPs	danger-associated molecular patterns
DNMT	DNA methyltransferase
DCs	myeloid dendritic cells (also dubbed ‘classical’ or ‘conventional’)
ECM	extracellular matrix
EGF	epidermal growth factor
EMT	epithelial-to-mesenchymal transition
FXR	farnesoid X receptor
GFAP	glial fibrillary acidic protein
HAND2	heart and neural crest derivatives 2
HBV	hepatitis B virus
HCC	hepatocellular carcinoma
HCV	hepatitis C virus
HDV	hepatitis D virus
HGF	hepatocyte growth factor
HMGB1	high mobility group box 1 protein
HNRNPA1	heterogeneous nuclear ribonucleoprotein A1
HSP47	Heat shock protein 47
ICAM-1	intercellular adhesion molecule 1
IL-6	interleukin 6
ILCs	innate lymphoid cells
KCs	Kupffer cells
lncRNAs	long noncoding RNAs
LPS	lipopolysaccharide
LRAT	lecithin retinol acyltransferase
LSEC	liver sinusoidal endothelial cells
MCP-1	monocyte chemoattractant protein-1
MDSCs	myeloid-derived suppressor cells
mito-DAMPs	mitochondria-derived danger signals
MMPs	matrix metalloproteinases
MOMP	mitochondrial outer membrane permeabilization
MSCs	mesenchymal stem cells
MSLN	mesothelin
MUCI 16	mucin 16
NAFLD	nonalcoholic fatty liver disease
NASH	nonalcoholic steatohepatitis
NETs	neutrophil extracellular traps
NKs	natural killer cells
NKTs	natural killer T cells
PAMPs	pathogen-associated molecular patterns
PBC	primary biliary cholangitis
PDGF	platelet-derived growth factor
PPARγ	peroxisome proliferator-activated receptor γ
PSC	primary sclerosing cholangitis
ROS	reactive oxygen species
SAMs	scar-associated macrophages
scRNA sequencing	single-cell RNA sequencing
SEREB-1c	sterol regulatory element-binding protein 1c
TET	methylcytosine dioxygenase
TGF-β	transforming growth factor-β
TIMPs	tissue inhibitors of metalloproteinases
TLR-4	Toll-like receptor 4
TRAIL	TNF-related apoptosis-inducing ligands
VCAM-1	vascular cell adhesion molecule 1
